# Dr. Freyer on His Operation for Enucleation of the Prostate

**Published:** 1902-02-22

**Authors:** 


### DR. FREYER ON HIS OPERATION FOR ENUCLEATION OF THE PROSTATE.

It is quite clear that at the present time a good deal of scepticism prevails among surgeons as to Dr. Freyer's operation for the total extirpation of the prostate. Not that there is any doubt about the brilliancy of the results obtained, for the cases related by Dr. Freyer are sufficient proof that the operation he describes is likely in certain conditions to be of immense utility, but rather as to the accuracy of Dr. Freyer's interpretation of what he does. The question lies in a nutshell. Does he enucleate and shell out of its bed—if it has a bed from which it can be thus enucleated and shelled out—the whole prostate gland in its own proper capsule, enclosing and carrying with it all the adenomatous masses by the growth of which it has become so greatly enlarged? which is what he says he does; or does he enucleate the adenomata alone, shelling them out of the compressed and shrivelled remains of the prostate gland which with the proper capsule are left in place? To the general practitioner, who merely sees in the operation a hopeful means of giving relief to a very grave condition, the search for an exact answer to this question may seem a matter of tweedledum and tweedledee. So that he knows to whom to send his case, he may not greatly care what are the exact anatomical relations of the "capsule" which is removed. But to surgeons it is a matter of considerable importance. As this question has come up again, we may properly refer to the remarks made by Dr. Freyer quite recently at a clinical lecture on the subject delivered at the Polyclinic. At a previous lecture he had given details of four cases in which he had successfully performed the operation of "total enucleation of the prostate in its capsule, the urethra being left behind intact," and he now proceeded to describe a further series of four cases in which he had undertaken the same operation, and very interesting these details are. "We have now," he says, "eight cases operated on, the ages varying from 62 to 76 years, all in broken health, and some of them almost in a dying condition before operation, with the prostates removed varying from $2\frac{1}{4}$ to $10\frac{1}{4}$ ounces. Seven of these have been completely successful in all respects. The remaining case had recovered from the operation and was passing his urine naturally, when he was seized by acute mania from which he died." Now as to what it is that he does in obtaining these results, Dr. Freyer is absolutely positive that what he removes is the entire gland in its capsule, and in proof of this he points to the specimens, saying, "you will observe that the growth is enveloped by a thin dense fibrous capsule which is closely adherent to it. This is the true capsule of the prostate." "There is much confusion in the text books," he says, "as to what constitutes the prostatic 'capsule,' some anatomists describing two distinct capsules—(1) a thin, strong, fibrous covering, closely adherent to the prostatic substance (this is the 'true capsule,' and that removed with the prostate in my operation), and (2) the fibrous casing outside this, containing the venous plexus formed by the recto-vesical fascia, and called the 'sheath' (which is left behind in my operation, and prevents infiltration of urine). Others, again, do not draw any distinction between these two, but regard the whole as the prostatic capsule. To persons brought up in this latter school my operation must necessarily appear absurd."

